# Vinculin, an adapter protein in control of cell adhesion signalling

**DOI:** 10.1016/j.ejcb.2010.06.007

**Published:** 2011-02

**Authors:** Alex Carisey, Christoph Ballestrem

**Affiliations:** Wellcome Trust Centre for Cell-Matrix Research, Faculty of Life Sciences, University of Manchester, Manchester, M13 9PT, UK

**Keywords:** Cell migration, Focal adhesion, Vinculin, Actin, Cell adhesion, Cell proliferation, Mechanical force

## Abstract

Vinculin, discovered in 1979 (Geiger, 1979), is an adapter protein with binding sites for more than 15 proteins. Biochemical and structural analyses have contributed to detailed knowledge about potential binding partners and the understanding of how their binding may be regulated. Despite all this information the molecular basis of how vinculin acts in cells and controls a wide variety of signals remains elusive. This review aims to highlight recent discoveries with an emphasis on how vinculin is involved in the coordination of a network of signals.

## Introduction

Vinculin is a 117 kDa protein, which localises to integrin-mediated cell–matrix adhesions and cadherin-mediated cell–cell junctions. In *C. elegans* vinculin deficiency leads to the loss of muscular activity and lethality at an early larval stage (Barstead and Waterston, 1989). Mouse embryos deficient in vinculin are small and die at day E10.5 with major defects in brain and heart development (Xu et al., 1998a). Embryos at E9.5 are about a third smaller than normal embryos and their mutant tissue seems more fragile suggesting that vinculin plays a role in strengthening cell attachments to its environment. Mouse embryonic fibroblasts isolated from vinculin knock-out animals at E9.5 spread less, have smaller focal adhesions (FA) and show decreased adhesion strength to fibronectin, laminin, vitronectin and collagen, but they migrate faster than their wild-type counterparts (Xu et al., 1998a). Earlier studies using vinculin-null F9 embryonic carcinoma cells made similar observations (Coll et al., 1995). Re-expression of vinculin rescued these defects in vinculin-null cells (Coll et al., 1995; Xu et al., 1998b; Saunders et al., 2006). The phenotypic changes of reduced cell adhesion and an increase in cell motility associated with the loss of vinculin is thought to drive the formation of tumour metastases. Other studies showed that expression of vinculin in tumour cell lines with diminished levels of the endogenous protein suppressed their tumorigenic ability and increased adhesion strength (Rodríguez Fernández et al., 1992; Lifschitz-Mercer et al., 1997). However it remains to be established which signals lead to the enhanced tumourigenicity of vinculin-deficient cells.

**Fig. 1:**
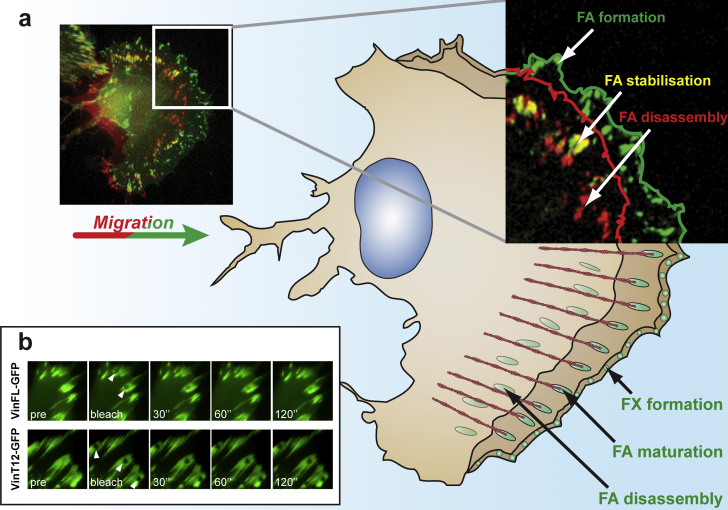
(a) Schematic representation originating from a migrating B16 melanoma cell expressing β3-integrin – GFP showing areas of FA formation and disassembly. To outline these areas, two images of time-lapse recordings taken at 5 min intervals were labeled in green and red respectively and then superimposed. Newly formed FA are labeled in green; structures that remained at the same place during the 5 min interval are seen in yellow; FA that have disassembled during cell motility in the later time point are labeled in red. Note that cell–matrix contacts start forming at the protrusive edge of the cells (focal complexes; FX), they then increase in size to mature into streak-shaped focal adhesions (FA), and then disassemble in an area before the nucleus. (b) Fluorescence Recovery After Photobleaching (FRAP) of vinculin-GFP fusion constructs in vinculin-deficient mouse embryonic fibroblasts (MEF). The top row shows the recovery of wild-type vinculin-GFP, the lower row the recovery of constitutively active vinculinT12-GFP in pre-bleached FA areas (see arrowheads). Note that the turnover of wild-type vinculin in FA is dramatically faster than the constitutively active vinculin T12 mutant.

During cell migration small dot-like adhesion sites (focal complexes; FX) form at the leading edge of a cell, which then mature into streak-shaped FA. In the lamellum, an area in front of the nucleus, adhesions start to disassemble (Fig. 1a). For controlled cell motility, the formation and disassembly of adhesion sites needs to be coordinated. However, it should be noted that FA do not only form during continuous cell migration, they can also be constantly formed in the cell periphery of stationary cells mostly at sites of local protrusions and retractions (Smilenov et al., 1999; Ballestrem et al., 2001; our observations). Although many of those FA, especially in less migratory cell types such as fibroblasts or epithelial cells, seem relatively stable over time, there is an astonishing level of mobility of proteins therein (Lele et al., 2008). Many FA proteins, including vinculin, can cycle in and out of these FA. The rate of vinculin cycling is dependent on its activity status (Fig. 1b), which in turn can affect the cycling rate of other FA proteins (Cohen et al., 2006; Humphries et al., 2007). Although, it is known that vinculin is recruited to FA very early during their development, little is known about the molecular basis of how these events are controlled. Similarly, even though it is known that the activation status of vinculin controls its mobility, we are far from understanding how this can control key signals in FA.

## Activation of vinculin

**Fig. 2:**
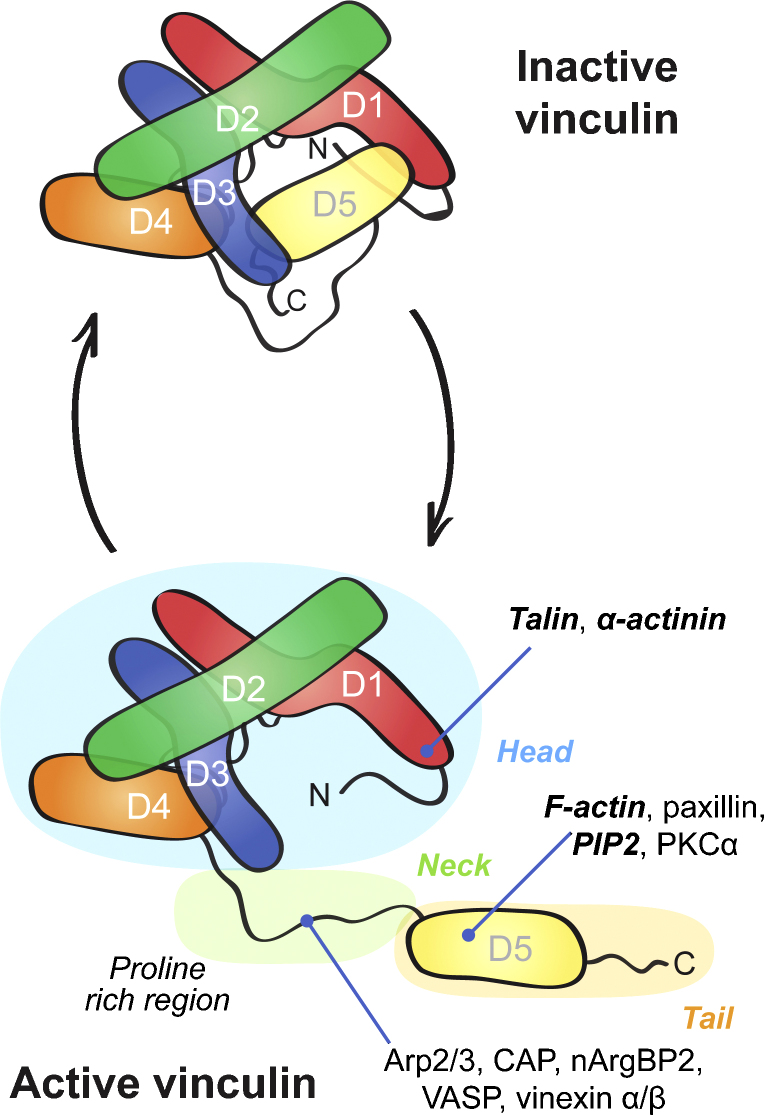
Schematic representation of vinculin cycling between active and inactive conformations. D1–D5 correspond to the individual domains of vinculin. D1–D4 assemble to form a globular head region which is connected by a flexible proline-rich neck to the vinculin tail, the D5 domain. A subset of binding partners that are thought to control together with vinculin the formation of FA are shown on the vinculin panel representing the active form. Partners involved in the activation of vinculin are in bold italic.

In 2005, Chen et al. were able to demonstrate that vinculin undergoes conformational changes when localising to FA (Chen et al., 2005). Using intramolecular Foerster Resonance Energy Transfer (FRET) they showed that only the active extended form of vinculin localises to focal adhesions whereas the folded inactive form resides in the cytoplasm. Evidence for such events was first obtained using biochemical and structural analyse of vinculin (Johnson and Craig, 1994) which revealed that vinculin is composed of head, neck and tail domains (Fig. 2) with the interaction between head and tail domains masking the binding sites for vinculin-binding partners (Ziegler et al., 2006). It is thought that, *in cellulo*, vinculin exists in an equilibrium between active and inactive states and it can be stabilised in the active form by interactions with a subset of binding partners. Several models for the activation of vinculin have been proposed all of which lead to its continued localisation to FA and the full unmasking of all binding sites. Again there is biochemical evidence from *in vitro* studies that talin or α-actinin, either as a single binding component (Izard et al., 2004; Izard and Vonrhein, 2004; Bois et al., 2006), or together with PIP2 (phosphatidylinositol-4,5-bisphosphate) or actin in what can be described as the combinatorial model (Bakolitsa et al., 2004; Chen et al., 2006), is able to disrupt the head–tail interaction and activate vinculin (Gilmore and Burridge, 1996; Izard et al., 2004; Bois et al., 2006; Janssen et al., 2006). Current evidence favours the combinatorial model since the tight intramolecular binding between the vinculin tail and head appears too strong for a single ligand to overcome (Janssen et al., 2006; Ziegler et al., 2006).

From studies in cell culture it seems likely that talin, rather than α-actinin, is the major protein involved in the activation of vinculin, since talin rather than α-actinin, co-localises with vinculin in FX (Zaidel-Bar et al., 2003). More importantly, the presence of talin is a prerequisite for vinculin localisation to FX (Zhang et al., 2008) and to FA (our unpublished observations). Whether PIP2 (Gilmore and Burridge, 1996; Weekes et al., 1996; Hüttelmaier et al., 1998) or actin (Chen et al., 2006) is the preferred partner for talin is yet unclear, but as the binding sites for actin and PIP2 overlap making it unlikely that both bind simultaneously (Steimle et al., 1999). A recent study has shown that vinculin mutants deficient in PIP2 binding readily localise to FA but inhibit FA turnover and subsequently decrease cell motility (Chandrasekar et al., 2005). The authors propose therefore that PIP2, rather than being involved in the activation of vinculin, regulates its release from FA plaque. One possibility is that PIP2 can compete and replace F-actin binding to vinculin (Chandrasekar et al., 2005). The precise role of actin in the activation process is also unclear and a number of questions arise. Would an initial contact with actin be sufficient to activate vinculin or are forces induced by the actomyosin machinery of the cell required for full activation? Evidence for the latter is that the expression of an isolated vinculin tail-GFP fusion protein was absent in large protrusions of cells and localised predominantly to actin stress fibres that seemed to be contracting (Humphries et al., 2007). But why full-length vinculin appears earlier than the isolated tail during focal adhesion assembly (Humphries et al., 2007) remains unclear. FRET studies showed that the active conformation is already present in FX (Chen et al., 2005) and it is known that FX can form without the requirement of actomyosin-dependent forces (Bershadsky et al., 1985; Ballestrem et al., 2001). More studies will be needed to elucidate the rather controversial evidence about the different activation mechanisms of vinculin in cells.

Although it is not clear whether paxillin also contributes to the activation of vinculin it seems to have some role in its recruitment to FA. Pasapera et al. (2010) recently proposed that myosin II-dependent forces activate FAK, which then phosphorylates paxillin on Y31 and Y118, leading to its association with vinculin. Testing the myosin II-dependence, cells treated with the inhibitor blebbistatin seemed to loose vinculin and FAK from FX, whereas paxillin appeared more stable. Expression of Y31/118E phosphomimetic paxillin was able to rescue vinculin recruitment into FX thus bypassing the apparent loss of paxillin phosphorylation by the reduction of FAK activity (Pasapera et al., 2010). However, controversially, other studies show that the highest phosphorylation state of paxillin is in FX (Zaidel-Bar et al., 2007b). These data would suggest that vinculin recruitment is highest in FX that form in a myosin II independent way. It was also shown that vinculin without its paxillin-binding tail domain can readily localise to FA and activates downstream paxillin recruitment in an indirect manner (Humphries et al., 2007). Moreover, an isolated tail domain bearing the paxillin-binding site does not localise to paxillin-positive FX and FA in protruding areas of cells (Humphries et al., 2007). These data imply that the binding site located in the tail of vinculin has little, if any, ability to bind paxillin directly. On the other hand one could argue that paxillin binding might need the full-length version of vinculin to be at the right time at the right place to interact locally with paxillin. Again more studies will be necessary to resolve the detailed relationship between paxillin and vinculin.

## The role of vinculin in the coordination of focal adhesion network

**Fig. 3:**
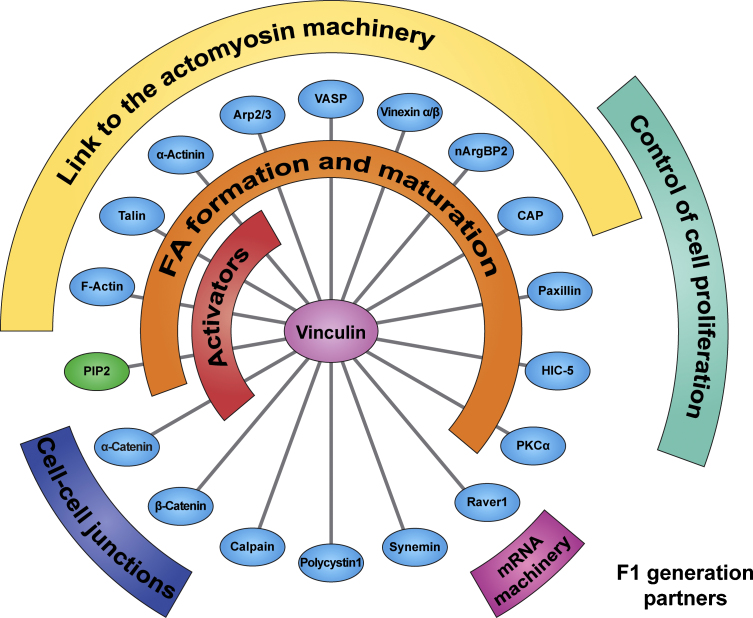
Representation of the direct binding partners of vinculin (the F1 generation, see text) linked to their reported functions. Partners in blue ovals indicate proteins; those in green ovals indicate phospholipids. The semi-circular areas outline the reported function as a consequence of vinculin binding to its individual partner.

The conversion of vinculin to an extended conformation allows full access of interacting partners to cryptic binding sites that are cryptic when it is inactive (Ziegler et al., 2006). To date 19 binding partners including talin (Gingras et al., 2005), α-actinin (Wachsstock et al., 1987), catenin α/β (Hazan et al., 1997; Watabe-Uchida et al., 1998), vinexin α/β (Kioka et al., 1999), CAP (c-Cbl-Associated Protein, also named ponsin or SH3P12) (Mandai et al., 1999), nArgBP2 (Kawabe et al., 1999), VASP (VAsodilator-Stimulated Phosphoprotein) (Brindle et al., 1996), Arp2/3 (Demali et al., 2002), paxillin (Wood et al., 1994), Hic-5 (Thomas et al., 1999), F-actin (Menkel et al., 1994; Hüttelmaier et al., 1997), PKCα (Ziegler et al., 2002), synemin (Bellin et al., 2001), calpain (Serrano and Devine, 2004), polycystin-1 (Geng et al., 2000), raver1 (Hüttelmaier et al., 2001) and PIP2 (Johnson et al., 1998) have been identified for vinculin. These directly interacting partners (F1 generation, Fig. 3) can, in turn, bind to other proteins (F2, F3 generation etc.) that link vinculin to a whole signalling network (Zaidel-Bar et al., 2007a). The potential number of F2 proteins affected by vinculin activity in F2 is more than 150 (not shown). However, it should be taken into account that some of these interactions, especially those that were characterised biochemically in experiments using peptides, may not occur *in cellulo*. Thus it is still important to elucidate which parts of the network are driven by vinculin activity in cells. The F1 interactions and their potential function discussed in this review are outlined in Fig. 3.

### Vinculin as a driving force behind focal adhesion formation

From studies using cells of vinculin knock-out mice it is clear that cell migration, as well as focal adhesion formation, is controlled by vinculin. More recently, we have shown that the interaction of vinculin with talin has a key role in regulating FA formation (Humphries et al., 2007). Expression of the N-terminal 258 amino acids (aa) of vinculin (vin258) was sufficient to promote dramatic growth of FA. Similar observations were made upon expression of a construct comprising the head and neck domain (first 880aa; vin880) and a constitutively active, full-length vinculin construct (vinT12; Cohen et al., 2005). All of these “active” constructs lost their FA growth-promoting activity when talin-binding was inhibited by mutation of its binding site in vinculin (A50I). Fluorescence Recovery After Photobleaching (FRAP) experiments (Fig. 1b) allowed us to analyse the effect of active vinculin on the dynamics of other proteins localising to FA. The decreased mobility of vinculin also decreased the turnover rate of talin (Cohen et al., 2006) and integrins in FA (Humphries et al., 2007). These findings led to the hypothesis that active vinculin, by forming a stable complex with talin and integrins, holds the adhesion receptors in a high affinity state thus promoting focal adhesion growth (Humphries et al., 2007).

### Role of vinculin controlling cell proliferation

One of the striking features of vinculin knock-out cells is that they become resistant to apoptotic signals (Subauste et al., 2004). This feature, together with other data showing the increased metastatic potential of cells lacking vinculin (Rodríguez Fernández et al., 1992), provides evidence for a role of vinculin in the regulation of cell survival and proliferation. At a molecular level there is evidence for two pathways indicating that vinculin may act as a tumour suppressor by inhibiting ERK (Extracellular signal-Regulated Kinase), a protein which has a key role in the regulation of cell proliferation.

One involves a role for vinculin in the modulation of the paxillin-FAK (Focal Adhesion Kinase) interaction (Subauste et al., 2004). Both vinculin knock-out cells and the expression of a construct with a mutation in the vinculin tail region (Y822F), showed an increase in FAK–paxillin interactions and phosphorylation. These in turn correlated with an up-regulation of ERK activity that suppressed apoptosis. The expression of only the neck–tail region of vinculin was able to restore the apoptotic potential. The phosphorylation of paxillin seemed to be crucial since expression of a paxillin Y31/118F dominant-negative mutant in vinculin knock-out cells inhibited ERK activation and restored sensitivity to apoptosis. These observations led to the proposal that the vinculin tail competes with FAK for paxillin binding thus regulating paxillin phosphorylation (Subauste et al., 2004).

The second vinculin-mediated pathway leading to ERK modulation is *via* the neck-binding protein CAP. The interaction of CAP with vinculin is crucial for its recruitment to focal adhesions (Zhang et al., 2006). Interestingly, CAP can also bind to FAK (Ribon et al., 1998) and paxillin (Zhang et al., 2006). However CAP, although possibly helping to enrich FAK and paxillin in FA, seems a negative regulator of ERK activity since its depletion led to enhanced fibronectin-mediated ERK activation (Zhang et al., 2006). This activation was not mediated by enhanced FAK phosphorylation but rather by the activation of PAK (p21-Activated Kinase) and MEK (MAP/ERK Kinase) upstream of ERK. Depletion of CAP also led to a 3-fold increase of cell motility. Whether this increase in cell motility correlates with increase in the metastatic potential in vinculin knock-out cells, which fail to recruit CAP to FA, remains to be investigated.

### Multiple links to the actin cytoskeleton

Vinculin has binding sites for actin and other proteins such as talin, α-actinin, VASP, Arp2/3, vinexin, nArgBP2 and CAP which can all associate with the actin cytoskeleton.

The direct interaction of vinculin with actin is mediated by two regions located in the tail (Hüttelmaier et al., 1997; Janssen et al., 2006). One of the regions appears to be exposed in the autoinhibited form of vinculin, the other one is hidden by the interaction with the head region (Janssen et al., 2006). Upon activation of vinculin its binding strength to actin increases, which, *in vitro*, leads to dimerisation of the vinculin tail (Johnson and Craig, 2000; Janssen et al., 2006) and actin bundling (Jockusch and Isenberg, 1981; Hüttelmaier et al., 1997). Interestingly, the isolated vinculin tail is monomeric and dimerises only when bound to F-actin (Johnson and Craig, 2000; Janssen et al., 2006), indicating that dimerisation occurs only after actin binding. Janssen et al. propose a model whereby actin binding of vinculin may weaken an interaction between the D4 region in the head domain and the tail domain (Fig. 2), which would then increase the likelihood for talin (in cell–matrix interactions) or α-catenin (in cell–cell adhesions) to bind to fully active vinculin by disrupting the interaction of the D1 domain (Fig. 2) and the vinculin tail (Janssen et al., 2006). It will be important to confirm such mechanisms in cells. A recent publication demonstrated the potential of the actin binding tail to trigger actin polymerisation (Wen et al., 2009) thereby suggesting that vinculin may act as an actin nucleation site in focal adhesions. Again work will be required to verify this finding in live cells.

Besides direct binding to actin, vinculin has interaction sites for proteins that are potent actin regulators; these proteins include Arp2/3 (Mullins et al., 1998), VASP (Reinhard et al., 1992) and members of the vinexin family (Kioka et al., 1999). The interaction of vinculin with the Arp2/3 complex is Rac-mediated and transiently present in FX during the process of cell spreading. Blocking of Arp2/3 binding to vinculin by mutation of P876 or P878 in vinculin inhibits efficient cell spreading and decreases cell migration (Demali et al., 2002). Both of these mutations were specific for Arp2/3 binding since they did not block CAP and VASP binding. It is not clear whether vinculin needs to be fully activated for the interaction.

The binding of vinculin to VASP is mediated by the first proline-rich region in the vinculin neck domain (Brindle et al., 1996). The interaction was greatly enhanced in PIP2-activated vinculin (Hüttelmaier et al., 1998) but the relevance of this interaction needs to be clarified especially since zyxin, another prominent adhesion component, has four proline-rich regions with stronger binding to VASP than vinculin (Reinhard et al., 1996). The close relationship of VASP with zyxin in cells has been outlined by Garvalov et al. (2003), showing that the presence of zyxin is a prerequisite for VASP recruitment to focal adhesions. Nevertheless, the formation of VASP tetramers (Zimmermann et al., 2002) could enable simultaneous binding of VASP to zyxin and vinculin; such interactions may form a strong actin nucleation nodule within FA.

The vinexin family members, vinexin α/β (Kioka et al., 1999), CAP (Mandai et al., 1999), and nArgBP2 (Cestra et al., 2005) bind to the proline-rich neck region of vinculin and have prominent roles in cytoskeletal organisation (reviewed in Kioka et al., 2002). For localisation of vinexin α/β and CAP to FA the presence of vinculin is essential (Chen et al., 2005; Takahashi et al., 2005; Zhang et al., 2006). CAP has been shown to bind directly to actin filaments and localised to actin stress fibres without its vinculin-binding SH3 domains (Zhang et al., 2006). Overexpression of CAP and α-vinexin leads to enhanced formation of stress fibres and reduced cell motility (Kioka et al., 1999; Takahashi et al., 2005; Zhang et al., 2006). Whether this phenotype is a result of its direct interaction with actin in cells, modulation of vinculin-F-actin binding, or other downstream signals, remains to be determined.

### The potential role of vinculin in transmission of mechanical forces

With its prominent links to the actin cytoskeleton, vinculin is a major candidate for mediating transmission of signals between the cell–matrix and the cytoskeleton, and there is accumulating evidence that vinculin regulates this process. Balaban et al. (2001) have shown that the levels of vinculin recruited to FA correlates directly to forces exerted to the matrix. Others have reported a high correlation in speed and direction of a dynamic flow from the cell periphery towards the nucleus of F-actin and vinculin; other FA plaque protein such as e.g. paxillin remained rather stationary (Hu et al., 2007). Our laboratory has shown that the dynamic retrograde flow of vinculin strongly depends on the presence of its actin binding tail. Without its actin binding tail, neither vinculin nor talin followed the retrograde flow of actin, exerted by the actomyosin machinery (Humphries et al., 2007; unpublished). These observations can be translated into a model whereby vinculin is placed between the integrin–talin complex and the actomyosin machinery and thus may be the major protein involved in the transduction of forces (Figs. 2 and 3). Such a model is supported by the detailed analysis of force fluctuations with the dynamic of F-actin and its coupling to vinculin (Ji et al., 2008). The model suggests that force transmission at focal adhesions requires the association of vinculin with F-actin and integrins.

Using vinculin knock-out MEF and RNAi knock-down, others have demonstrated that vinculin contributes approximately 30% to the adhesion strength of cells upon generation of a 200 nN adhesive force (Gallant et al., 2005). More recently the same group demonstrated that FAK regulates adhesive forces by modulating vinculin localisation. In the presence of vinculin a reduction of FAK in focal adhesions led to increased vinculin levels and increased binding strength of the cell to ECM. Knock-down of vinculin eliminated increase in binding (Dumbauld et al., 2009).

Overall these data suggest that vinculin indeed has an important role in the transduction of mechanical forces towards the actin cytoskeleton initially sensed by the cell *via* integrin-binding to ECM proteins.

## Vinculin in cell–cell contacts

There is clear evidence for vinculin localisation at cell–cell junctions (Geiger et al., 1980, 1981; Rüdiger, 1998). Although vinculin is not essential for the formation of cell–cell junctions, deficiency or reduced levels can lead to junctions that are not fully functional (Watabe-Uchida et al., 1998; Maddugoda et al., 2007). Thus it is thought that vinculin might enforce mechanical links between the cell–cell adhesion complex and the actin cytoskeleton analogous to events during FA formation. In adherens junctions cell–cell adhesion receptors (E-cadherins) first cluster at tips of actin rich cell protrusions (filopodia) generating initial adhesion complexes when opposing cells come into contact. These initial contacts, whose formation was dependent on the presence of α-catenin, also contain vinculin (Vasioukhin et al., 2000). α-Catenin can interact with the N-terminal head region of vinculin (Watabe-Uchida et al., 1998; Weiss et al., 1998) and the C-terminal tail of vinculin can substitute in a fusion with the N-terminal part of α-catenin the link of the junctional complex to actin. The same study demonstrated that the impaired junctional complex organisation in vinculin-deficient F9 cells could be rescued by the re-expression of vinculin (Watabe-Uchida et al., 1998). Thus, although α-catenin itself has a binding site for actin, bridging the link to actin *via* vinculin may strengthen this link.

Also β-catenin was reported to bind vinculin and it was shown that in cells lacking α-catenin, the β-catenin-vinculin interaction was required for cadherin-mediated cell–cell adhesion complex formation (Hazan et al., 1997). In a recent study, Peng and colleagues (Peng et al., 2010) suggested that vinculin binding to β-catenin stabilises E-cadherin on the cell surface, thereby regulating its expression. Also myosin VI is part of a complex that together with vinculin cooperates in regulating E-cadherin-mediated formation of cell–cell contacts (Maddugoda et al., 2007). The exact molecular basis of how this complex is formed is still unknown but the authors hypothesise that myosin VI and vinculin cooperate to form the perijunctional actin cytoskeleton leading to a strengthening of cell–cell contacts. Interestingly, others recently published evidence of a tension-modulated recruitment of vinculin in adherens junctions occurring through the stretching of α-catenin (Yonemura et al., 2010). Force-dependent changes in α-catenin conformation unmask binding sites for vinculin, leading to the anchorage of more actin fibres to this adherens junction. This is thought to counterbalance an increased tension between cells with a more robust actin cytoskeleton.

## Vinculin as a scaffold for mRNA translation

Raver1 is a member of the heterogeneous nuclear ribonucleoprotein family (hnRNP) that shuttles between the nucleus and cytoplasm and is involved in modulation of alternative splicing mediated by PTB (Polypyrimidine Tract-Binding protein) (Gromak et al., 2003). The reported interaction between raver1 and vinculin (Hüttelmaier et al., 2001) suggests a role for vinculin as a docking station for complexes involved in mRNA processing. Analysis of the crystal structure of the complex (Lee et al., 2009) has shown that raver1 interacts with the vinculin tail domain after vinculin activation potentially enabling local translation of focal adhesion plaque proteins (Madl and Sattler, 2009). This model is supported by the observation that raver1 can drive vinculin mRNA to FA through a specific RNA binding sequence allowing *in situ* expression and local delivery of protein. Vinculin activation may play also a role in targeted mRNA delivery to specific sites. It remains to be demonstrated how important this local delivery of mRNA in context with the raver1-vinculin interaction is, especially since knock-out of raver1 shows little, if any, phenotype (Lahmann et al., 2008).

## Future outlook

Vinculin is one of the best characterised protein of the adhesion complex. Despite important biochemical and structural data, the understanding of how vinculin contributes to the organisation of an entire signalling network is far from being resolved. Vinculin activation pathways remain controversial and the knowledge of how vinculin controls the adhesion network in cell–matrix and cell–cell adhesions is rather fragmented. Although much detailed information has been reported on vinculin interactions, we have no holistic view of how vinculin coordinates the vast variety of signals. Advances in technology will enable us to achieve a clearer understanding of vinculin function. Conditional knock-out experiments will support a better comprehension of the role of vinculin in a variety of tissues; advances in mass spectrometry technologies offer the potential to study hierarchies of protein binding to vinculin in cellular systems; advanced imaging, including image correlation analysis, analysis of protein dynamics and high resolution imaging and FRET, should help us map vinculin localisation and interactions in time and space. These techniques, together with bioinformatics and mathematical modelling could bring us closer to solving how vinculin orchestrates adhesion systems.
